# Renal and renal sinus fat volumes as quantified by magnetic resonance imaging in subjects with prediabetes, diabetes, and normal glucose tolerance

**DOI:** 10.1371/journal.pone.0216635

**Published:** 2020-02-19

**Authors:** Mike Notohamiprodjo, Martin Goepfert, Susanne Will, Roberto Lorbeer, Fritz Schick, Wolfgang Rathmann, Petros Martirosian, Annette Peters, Katharina Müller-Peltzer, Andreas Helck, Susanne Rospleszcz, Fabian Bamberg

**Affiliations:** 1 Department for Diagnostic and Interventional Radiology, University Hospital Tuebingen, Tuebingen, Germany; 2 DIE RADIOLOGIE, Munich, Germany; 3 Department of Radiology, University Hospitals Munich, Munich, Germany; 4 German Center for Cardiovascular Disease Research, Munich, Germany; 5 Institute for Biometrics and Epidemiology, German Diabetes Center, Duesseldorf, Germany; 6 German Center for Diabetes Research (DZD), München-Neuherberg, Germany; 7 Institute of Epidemiology, Helmholtz Zentrum München, German Research Center for Environmental Health, Neuherberg, Germany; 8 Chair of Epidemiology, Ludwig-Maximilians-University Munich, Munich, Germany; 9 Department of Diagnostic and Interventional Radiology, Medical Center - University of Freiburg, Freiburg, Germany; University Medical Center Utrecht, NETHERLANDS

## Abstract

**Purpose:**

We hypothesize that MRI-based renal compartment volumes, particularly renal sinus fat as locally and potentially independently acting perivascular fat tissue, increase with glucose intolerance. We therefore analyze the distribution of renal volumes in individuals with normal glucose levels and prediabetic and diabetic individuals and investigate potential associations with other typical cardiometabolic biomarkers.

**Material and methods:**

The sample comprised N = 366 participants who were either normoglycemic (N = 230), had prediabetes (N = 87) or diabetes (N = 49), as determined by Oral Glucose Tolerance Test. Other covariates were obtained by standardized measurements and interviews. Whole-body MR measurements were performed on a 3 Tesla scanner. For assessment of the kidneys, a coronal T1w dual-echo Dixon and a coronal T2w single shot fast spin echo sequence were employed. Stepwise semi-automated segmentation of the kidneys on the Dixon-sequences was based on thresholding and geometric assumptions generating volumes for the kidneys and sinus fat. Inter- and intra-reader variability were determined on a subset of 40 subjects. Associations between glycemic status and renal volumes were evaluated by linear regression models, adjusted for other potential confounding variables. Furthermore, the association of renal volumes with visceral adipose tissue was assessed by linear regression models and Pearson’s correlation coefficient.

**Results:**

Renal volume, renal sinus volume and renal sinus fat increased gradually from normoglycemic controls to individuals with prediabetes to individuals with diabetes (renal volume: 280.3±64.7 ml vs 303.7±67.4 ml vs 320.6±77.7ml, respectively, p < 0.001). After adjustment for age and sex, prediabetes and diabetes were significantly associated to increased renal volume, sinus volume (e.g. β_Prediabetes_ = 10.1, 95% CI: [6.5, 13.7]; p<0.01, β_Diabetes_ = 11.86, 95% CI: [7.2, 16.5]; p<0.01) and sinus fat (e.g. β_Prediabetes_ = 7.13, 95% CI: [4.5, 9.8]; p<0.001, β_Diabetes_ = 7.34, 95% CI: [4.0, 10.7]; p<0.001). Associations attenuated after adjustment for additional confounders were only significant for prediabetes and sinus volume (ß = 4.0 95% CI [0.4, 7.6]; p<0.05). Hypertension was significantly associated with increased sinus volume (β = 3.7, 95% CI: [0.4, 7.0; p<0.05]) and absolute sinus fat volume (β = 3.0, 95% CI: [0.7, 5.3]; p<0.05). GFR and all renal volumes were significantly associated as well as urine creatinine levels and renal sinus volume (β = 1.6, 95% CI: [0.1, 2.9]; p<0.05).

**Conclusion:**

Renal volume and particularly renal sinus fat volume already increases significantly in prediabetic subjects and is significantly associated with VAT. This shows, that renal sinus fat is a perivascular adipose tissue, which early undergoes changes in the development of metabolic disease. Our findings underpin that renal sinus fat is a link between metabolic disease and associated chronic kidney disease, making it a potential imaging biomarker when assessing perivascular adipose tissue.

## Introduction

Parenchymal abnormalities of the kidneys are closely linked to the development and outcome of cardiovascular disease [1–6]. The exact mechanisms that link renal abnormalities in obesity with cardiovascular complications, insulin resistance, type 2 diabetes and hypertension are still not sufficiently understood. The Framingham Study has shown that increasing total kidney volume is associated with diabetes [7] and that furthermore renal sinus fat is associated to cardiometabolic risk factors [8], making a potential interacting and factor between renal abnormality and systemic multiorgan disease. As perivascular fat depot it is in close contact with the adventitia of large, medium and small arteries and possesses unique features differing from other fat depots. Renal sinus fat is apparently directly linked to obesity [9] as well as liver function [10] and is thought to obstruct the blood and lymph outflow of the kidney, thus increasing parenchymal hydrostatic pressure leading to increasing organ volume [11]. Similarly, to other perivascular fat tissue particularly intrahepatic fat and visceral adipose tissue (VAT), it is associated with cardiovascular risk factors such as hypertension, diabetes and chronic kidney disease [11–13]. Quantification of renal sinus fat may yield a biomarker for early morphological changes in diabetic nephropathy and may further help in phenotyping the extent of cardiometabolic syndrome.

Magnetic resonance imaging allows for excellent anatomical separation without the need of radiation or contrast agent administration. Multi-Echo sequences provide comprehensive image contrast, so that an in-phase, opposed-phase, fat only and water image is provided [14]. This combination of contrasts allows for improved segmentation of the renal compartments. This methodology also forms the basis for absolute MR-quantification of renal sinus and intrarenal adipose tissue [15, 16]. A semi-automated approach may yield a reliable method for high through-put quantification of the kidney compartments for large scale cohort studies.

Whole-body MRI has been implemented in population-based studies to detect early disease stages and imaging biomarkers indicative of increased risk of developing diseases in the future. Whole-body MRI has been implemented in population-based studies to detect early disease stages and imaging biomarkers indicative of increased risk of developing diseases in the future. A clinically well characterized subset of participants from the KORA cohort (Kooperative Gesundheitsforschung in der Region Augsburg) have undergone whole-body MRI for detection of phenotypical changes associated with cardiometabolic disease, such as liver fat, VAT or pancreatic fat [17, 18].

We hypothesize that MRI-based renal compartment volumes, particularly renal sinus fat as locally and potentially independently acting perivascular fat tissue, increase with glucose intolerance. We therefore analyze the distribution of renal volumes in individuals with normal glucose levels and prediabetic and diabetic individuals and investigate potential associations with other typical cardiometabolic biomarkers.

## Material and methods

### Study design

The study population consisted of a cross-sectional subsample of N = 400 whole-body MR participants from the population-based KORA FF4 cohort from the region of Augsburg, Germany. KORA FF4 (N = 2279, enrolled in 2013/2014) is the second follow-up of the original S4 survey (N = 4261, enrolled in 1999–2001, first follow-up: F4, N = 3080, enrolled in 2006–2008) [19]. The setup of the MR substudy in KORA FF4 has been described previously [17]. Eligible subjects were selected if they met the following inclusion criteria: willingness to undergo whole-body MRI; and qualification as being in the prediabetes, diabetes, or control group (see Covariate Assessment). Exclusion criteria were: age >72 years, subjects with validated/self-reported stroke, myocardial infarction, or revascularization; subjects with a cardiac pacemaker or implantable defibrillator, cerebral aneurysm clip, neural stimulator, any type of ear implant, an ocular foreign body, or any implanted device; pregnant or breast-feeding subjects; and subjects with claustrophobia, known allergy to gadolinium compounds, or serum creatinine level ≥ 1.3 mg/dl [17].

Data on VAT and intrahepatic fat was also derived from the same study. The study cohort has been analyzed in several other manuscripts [17, 18, 20–23]. For detailed information please refer to the respective manuscripts.

This study was approved by the institutional review board of the Ludwig Maximilian’s University Munich (Germany) and written consent was obtained from each participant.

### MR imaging protocol

Whole-body MR measurements were performed on a 3 Tesla scanner (Magnetom Skyra, Siemens Healthcare, Erlangen, Germany). Detailed descriptions of technical and imaging protocols are listed elsewhere [17]. For assessment of the kidneys, a coronal T1w dual-echo Dixon and a coronal T2w single shot fast spin echo (SS-FSE/HASTE) sequence were employed. Imaging parameters dual-echo Dixon: 256 x 256, field of view (FOV): 488 x 716 mm, echo time (TE) 1.26 ms and 2.49 ms, repetition time (TR): 4.06 ms, partition segments: 1.7 mm, flip angle: 9°. Image parameter T2 Haste: matrix: 320 x 200, field of view (FOV): 296 x 380 mm, echo time (TE) 91 ms, repetition time (TR): 1000 ms, partition segments: 5 mm, flip angle: 131°.

### Kidney segmentation

**Fig 1:**
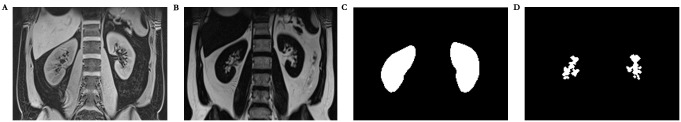
Exemplary segmentation of a coronal T1w Dixon-VIBE-dataset (A). Corresponding fat only images (B). A whole kidney mask is generated using thresholding and active contours (C). Next the renal sinus fat is segmented using thresholding of fat isointense voxels (D).

The semi-automated image segmentation was performed using Matlab (Version R2011b, The MathWorks, Natick, USA) (Fig 1) and is based on the algorithm described in [24]. Similar methodology has been used analysis of the Framingham Heart Study and is considered the standard of reference for MR-based kidney volumetry [7]. In short, the kidneys were segmented from the surrounding tissues by thresholding the Dixon-T1 water-only images with a subsequent refinement step using prior knowledge about the kidney shape and location. In a second step renal parenchyma, renal sinus and sinus fat were determined by thresholding the maximum pixel’s intensity in the slice. The separation of the kidney from the spleen and gastrointestinal tract was refined using active contours generating a whole kidney mask. Within the generated entire kidney mask, the kidney, renal sinus and pelvis were subsequently separated. This separation algorithm utilizes assumptions of the renal anatomical structure, e.g., that the renal cortex surrounds parts of the medulla. The renal sinus was segmented using pixels with lower signal intensity than renal parenchyma tissue in water-only T1w- images. The fat-only pixels were identified through their position and separated from the pelvis mask. Afterwards, the union pelvis mask was subtracted from the kidney mask to separate kidney, renal sinus and pelvis. The resulting masks of the respective compartments were inspected by one reader (M.G.) and manually corrected by eliminating voxels mistakenly considered as renal parenchyma, mostly from the liver or spleen. The final volumes of the entire kidneys, renal cortex, medulla, and pelvis were then calculated by voxel summation. A subset of 33 study participants was also evaluated by a second reader (S.W.) for assessment of inter-reader variability.

### Covariate assessment

Trained staff obtained covariates by standardized interviews and standard laboratory tests. Oral Glucose Tolerance Test (OGTT) was used to determine glycemic status as normoglycemic control, prediabetes or diabetes according to WHO criteria: Prediabetes was determined by normal fasting glucose concentration and a 2-h serum glucose concentration ranging between 140 and 200 mg/dl; and/or an impaired fasting glucose concentration, as defined by fasting glucose levels between 110 and 125 mg/dl, and a normal 2-h serum glucose concentration. Diabetes was determined by a 2-h serum glucose concentration >200 mg/dl and/or a fasting glucose level > 125 mg/dl. Normoglycemic controls was determined by normal glucose metabolism with a 2-h serum glucose concentration <140 mg/dl and a fasting glucose level that was <110 mg/dl [17]. Enzymatic, colorimetric assays were used to measure cholesterol value, while albumin was measured by an immunonephelometric assay [25]. GFR was calculated from serum creatinine using the CKD-EPI definition [26], stratified by sex. Hypertension was defined as systolic blood pressure > = 140 mmHg or diastolic blood pressure > = 90 mmHg or intake of antihypertensive medication while being aware of having hypertension.

### Statistical analysis

Demographic data, covariates and MRI-derived renal volumes, VAT and intrahepatic fat are presented as arithmetic means with standard deviation for continuous variables and counts with percentages for categorical variables.

Associations between glycemic status and renal volumes were determined by linear regression adjusted for additional potential confounding covariates. Regression coefficients β with corresponding 95% Confidence Intervals (CI) and p-values are reported. Furthermore, correlations between renal volumes and VAT were explored graphically by scatterplots and calculated quantitatively by Pearson’s correlation coefficient. Associations were determined by linear regression models; the corresponding R^2^ served as a measure of how much variance in the outcome was explained by the model.

Inter-reader variability was determined by calculating the relative and absolute difference for derived values, as well as calculation of the intraclass correlation coefficient (ICC).

All calculations were conducted with R v3.3.1. P-values < 0.05 are considered to denote statistical significance.

## Results

### Study subjects

**Fig 2:**
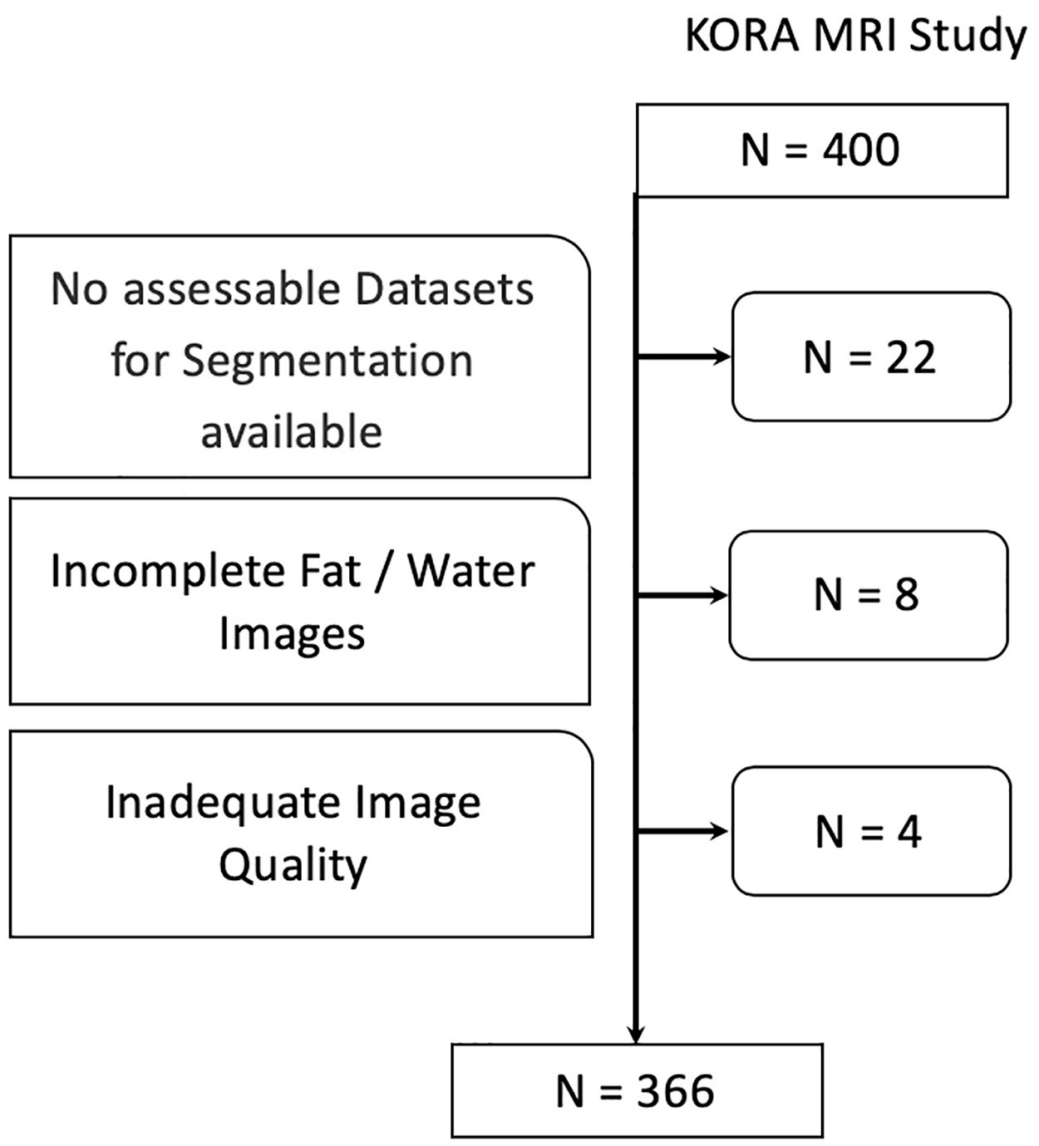
Inclusion flow chart. N = 366 study subjects were finally included for analysis.

Detailed information is available in Table 1. Among the 400 participants of the KORA-MRI Study, 366 (92%) subjects were included. Twenty-two (5.5%) subjects met the exclusion criteria due to none assessable datasets (9.3%), incomplete fat / water images (2%) or inadequate image quality (1%). Of the included subjects, 49 (13.4%) had diabetes and 87 (23.8%) prediabetes while 230 (62.8%) were normoglycemic. (Fig 2).

**Table 1:** Demographics, cardiovascular risk factors and MRI parameters of the study participants.

	All	Control	Prediabetes	Diabetes
	N = 366	N = 230 (62.8%)	N = 87 (23.8%)	N = 49 (13.4%)
Age, years	56.2 ± 9.1	54.4 ± 8.9	58.1 ± 8.6	61.4 ± 8.3
Male, %	208 (56.8%)	117 (50.9%)	55 (63.2%)	36 (73.5%)
Weight, kg	82.9 ± 16.8	78.2 ± 15.5	91.5 ± 14.9	89.5 ± 17.9
Height, cm	171.5 ± 9.8	171.2 ± 10.3	172.3 ± 9.2	171.3 ± 7.9
BMI, kg/m2	28.1 ± 5.0	26.6 ± 4.3	30.9 ± 5.0	30.4 ± 5.2
Waist circumference, cm	98.5 ± 14.6	93.5 ± 12.8	106.6 ± 13.0	107.8 ± 14.5
Waist-To-Hip-Ratio	0.9 ± 0.1	0.9 ± 0.1	1.0 ± 0.1	1.0 ± 0.1
Systolic Blood Pressure, mmHg	120.8 ± 16.9	116.6 ± 15.2	125.8 ± 15.1	131.9 ± 20.4
Diastolic Blood pressure, mmHg	75.3 ± 10.1	73.6 ± 9.2	78.4 ± 9.4	78.1 ± 13.1
Hypertension	124 (33.9%)	48 (20.9%)	41 (47.1%)	35 (71.4%)
Antihypertensive medication	91 (24.9%)	38 (16.5%)	29 (33.3%)	24 (49.0%)
Smoking				
Never-smoker	134 (36.6%)	90 (39.1%)	27 (31.0%)	17 (34.7%)
Ex-smoker	160 (43.7%)	91 (39.6%)	45 (51.7%)	24 (49.0%)
Smoker	72 (19.7%)	49 (21.3%)	15 (17.2%)	8 (16.3%)
Total Cholesterol, mg/dl	218.2 ± 37.1	216.1 ± 36.2	225.5 ± 32.0	214.9 ± 47.3
HDL Cholesterol, mg/dl	61.8 ± 18.0	65.4 ± 18.1	57.6 ± 14.5	51.9 ± 18.4
LDL Cholesterol, mg/dl	140.3 ± 33.4	138.5 ± 32.2	147.6 ± 30.1	136.1 ± 42.1
Triglycerides, mg/dl	131.5 ± 86.6	106.9 ± 64.3	153.6 ± 83.4	207.9 ± 123.2
GFR (CKD-EPI)	92.9 ± 13.0	94.9 ± 12.5	89.3 ± 12.2	89.6 ± 14.8
Urine Albumin, mg/l	25.3 ± 130.4	10.2 ± 15.8	13.5 ± 19.1	119.2 ± 345.1
Urine Creatinine, g/L	1.6 ± 0.8	1.6 ± 0.8	1.6 ± 0.7	1.8 ± 0.8
UACR, mg/g	15.4 ± 75.4	7.0 ± 10.1	8.3 ± 11.3	68.3 ± 199.6
Visceral Fat, l	4.5 ± 2.7	3.5 ± 2.3	5.8 ± 2.3	6.8 ± 2.4
Hepatic fat (PDFF), %	8.6 ± 7.7	5.6 ± 5.1	12.3 ± 7.8	16.1 ± 9.3
Renal Volume, ml	291.3 ± 68.7	280.3 ± 64.7	303.7 ± 67.4	320.6 ± 77.7
Sinus Volume, ml	40.0 ± 18.0	34.6 ± 16.0	47.6 ± 16.2	52.0 ± 19.4
Sinus Fat, ml	26.2 ± 13.6	22.2 ± 12.4	32.0 ± 12.0	34.5 ± 14.1

HDL, high-density lipoprotein; LDL, low-density lipoprotein; GFR, glomerular filtration rate, PDFF, proton density fat fraction; UACR, urin albumin to creatinine ratio

Subjects with prediabetes and diabetes had increasing cardiovascular risk factors and metabolic syndrome components waist circumference, weight, BMI, blood pressure, blood lipids, pericardial fat and VAT. GFR showed a slight but significant decline between groups. There was no significant difference for blood albumin.

### Inter-reader-variability

Inter-Reader Variability was evaluated on 33 subjects. The relative difference between readers for absolute renal volume was -1.9 ml (corresponds to -0.5%, 95% limits of agreement: -29.1 ml, 25.2 ml), whereas the relative difference for renal sinus volume was -5.1 ml (corresponds to -15.2%, 95% limits of agreement: -23.3 ml, 13.0 ml) and for the percentage of renal sinus fat 7.1% (corresponds to 14.7%, 95% limits of agreement: -8.3%, 22.5%).

Inter-reader-variability was assessed using intraclass correlation coefficients (ICC) from two-way random-effects ANOVA. An ICC value close to 1 indicates excellent agreement between the two observers. Absolute Renal Volume: 0.97 (0.92;0.99); Renal Sinus Volume: 0.99 (0.96;0.99); Renal Sinus Fat: 0.97 (0.95;0.99).

### Unadjusted renal volumes

**Fig 3:**
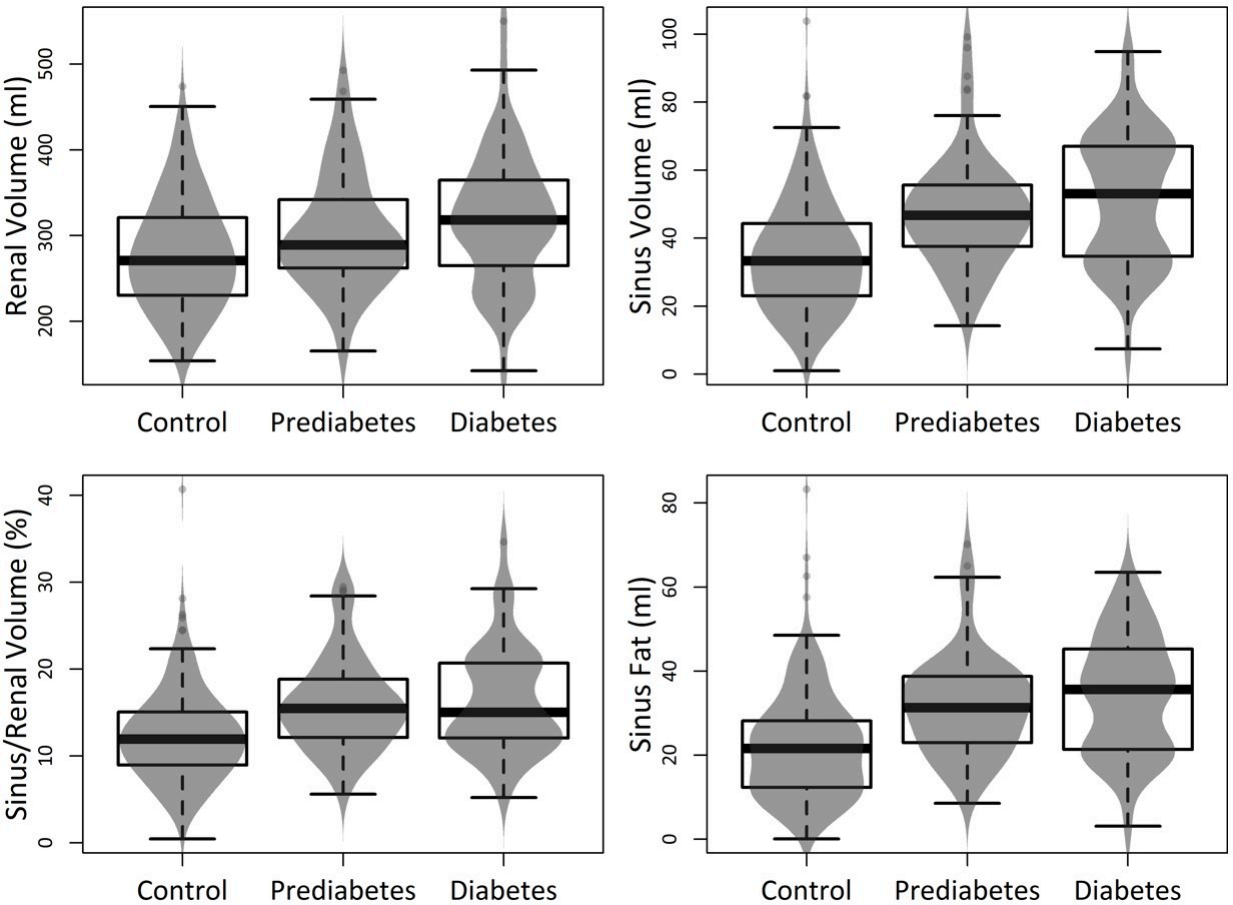
Boxplots with density curves displaying the distribution of renal and sinus fat volumes according to glycemic status. There was a considerable increase between controls and subjects with prediabetes particularly for renal sinus fat.

Detailed information is provided in Table 1 and Fig 3. Average renal volume showed a slight but highly significant increase between normoglycemic individuals and subjects with prediabetes and diabetes. Also, the renal sinus showed a significant enlargement between normoglycemic individuals and subjects with prediabetes and diabetes (renal volume: 280.3±64.7 ml vs 303.7±67.4 ml vs 320.6±77.7ml, respectively, p < 0.001). The largest difference was found between normoglycemic subjects and subjects with prediabetes (p<0.001 respectively). The sinus fat component showed very similar changes.

### Adjustment for age and sex

After adjustment for age and sex, a significant association could be found for prediabetes and diabetes with renal volume (Table 2). A prediabetic status was significantly associated with increased sinus volume (β = 10.08, 95% CI: [6.5, 13.7]; p<0.01) and sinus fat (β = 7.13, 95% CI: [4.5, 9.8]; p<0.001). Diabetes was also significantly associated with increased sinus volume (β = 11.86, 95% CI: [7.2, 16.5]; p<0.01) and sinus fat (β = 7.34, 95% CI: [4.0, 10.7]; p<0.001). Increasing age was significantly associated with decreasing kidney volume (β = -1.35., 95% CI: [-2.0, 0.7]; p<0.01).

**Table 2:** Regression model with adjustments for age, gender and glycemic status.

	Renal Volume (ml)			Sinus Volume (ml)			Sinus Fat Component (ml)		
	β	95%-CI	P-Value	β	95%-CI	P-Value	β	95%-CI	P-Value
Age, Years	-1.35	[-2.0, -0.7]	<0.001	0.25	[0.1, 0.4]	0.003	0.27	[0.1, 0.4]	<0.001
Sex, female	-79.04	[-90.3, -67.8]	<0.001	-16.58	[-19.6, -13.6]	<0.001	-13.95	[-16.1, -11.8]	<0.001
Prediabetes	18.65	[5.2, 32.1]	0.007	10.08	[6.5, 13.7]	<0.001	7.13	[4.5, 9.8]	<0.001
Diabetes	31.90	[14.6, 49.2]	<0.001	11.86	[7.2, 16.5]	<0.001	7.34	[4.0, 10.7]	<0.001
	R^2^_adj_ = 0.39915			R^2^_adj_ = 0.36517			R^2^_adj_ = 0.41438		

### Adjustment for variables associated with metabolic syndrome

After adjustment for VAT, HDL, LDL, urine albumin, liver fat, GFR and hypertension the association between glycemic status and renal volumes decreased and was only significant for prediabetes and sinus volume (Table 3) (ß = 4.0 95% CI [0.4, 7.6]; p<0.05). Hypertension was significantly associated with increased sinus volume (β = 3.7, 95% CI: [0.4, 7.0; p<0.05]) and absolute sinus fat volume (β = 3.0, 95% CI: [0.7, 5.3]; p<0.05). GFR and all renal volumes were significantly associated as well as urine creatinine levels and renal sinus volume (β = 1.6, 95% CI: [0.1, 2.9]; p<0.05).

**Table 3:** Regression model with adjustments for age, VAT, HDL, LDL, urine albumin/creatinine, liver fat, GFR, gender, hypertension yes/no and glycemic status.

	Renal Volume (ml)			Sinus Volume (ml)			Sinus Fat Component (ml)		
	β	95%-CI	P-Value	β	95%-CI	P-Value	β	95%-CI	P-Value
Age, Years	0.7	[-6.3, 7.7]	0.844	2.5	[0.7, 4.4]	0.006	2.3	[1.0, 3.6]	0.000
VAT	10.1	[1.6, 18.7]	0.020	7.6	[5.4, 9.8]	0.000	6	[4.5, 7.6]	0.000
HDL	-10.5	[-16.8, -4.2]	0.001	0.2	[-1.4, 1.9]	0.785	-0	[-1.2, 1.1]	0.959
LDL	-7.6	[-13.0, -2.2]	0.006	-1.4	[-2.8, 0.0]	0.055	-0.5	[-1.5, 0.5]	0.347
UACR	1.9	[-3.5, 7.3]	0.492	1.5	[0.1, 2.9]	0.034	0.3	[-0.7, 1.3]	0.538
Liver Fat	0.3	[-7.1, 7.6]	0.946	1.6	[-0.3, 3.5]	0.095	1.6	[0.3, 2.9]	0.018
GFR	23.1	[16.4, 29.7]	0.000	3	[1.2, 4.7]	0.001	2.1	[0.9, 3.3]	0.001
Sex, female	-57.6	[-70.6, -44.6]	0.000	-8.8	[-12.2, -5.5]	0.000	-7.3	[-9.7, -5.0]	0.000
Hypertonia, yes	10.2	[-2.5, 22.9]	0.115	3.7	[0.4, 7.0]	0.026	3	[0.7, 5.3]	0.011
Prediabetes	11.2	[-2.7, 25.1]	0.113	4	[0.4, 7.6]	0.030	1.7	[-0.8, 4.2]	0.177
Diabetes	7.9	[-11.0, 26.7]	0.413	-0	[-4.9, 4.8]	0.992	-1.6	[-5.0, 1.8]	0.357
	R^2(adj) = 0.498			R^2(adj) = 0.52002			R^2(adj) = 0.58631		

VAT, visceral adipose tissue; HDL, high-density lipoprotein; LDL, low-density lipoprotein; GFR, glomerular filtration rate, PDFF, proton density fat fraction; UACR, urine albumin to creatinine ratio

### Association and correlation between renal volumes and VAT

There was a highly significant association between VAT and renal volumes, particularly between VAT and the absolute sinus fat volume (β = 2.75, 95% CI: [2.3, 3.2]; p<0.01) (Table 3). A regression model only adjusted for age, sex and age already accounts for 55.6% of the variability of sinus fat (Table 4).

**Table 4:** Regression model with adjustments for age, gender and VAT.

	Renal Volume (ml)			Sinus Volume (ml)			Sinus Fat Component (ml)		
	β	95%-CI	P-Value	β	95%-CI	P-Value	β	95%-CI	P-Value
Age, Years	-1.53	[-2.2, -0.9]	<0.001	0.13	[-0.0, 0.3]	0.096	0.15	[0.0, 0.3]	0.006
VAT	6.06	[3.6, 8.6]	<0.001	3.57	[2.9, 4.2]	<0.001	2.75	[2.3, 3.2]	<0.001
Sex, female	-66.35	[-79.4, -53.3]	<0.001	-8.3	[-11.5, -5.1]	<0.001	-7.43	[-9.7, -5.2]	<0.001
	R^2^_adj_ = 0.41463			R^2^_adj_ = 0.48			R^2^_adj_ = 0.55612		

VAT, visceral adipose tissue

**Fig 4:**
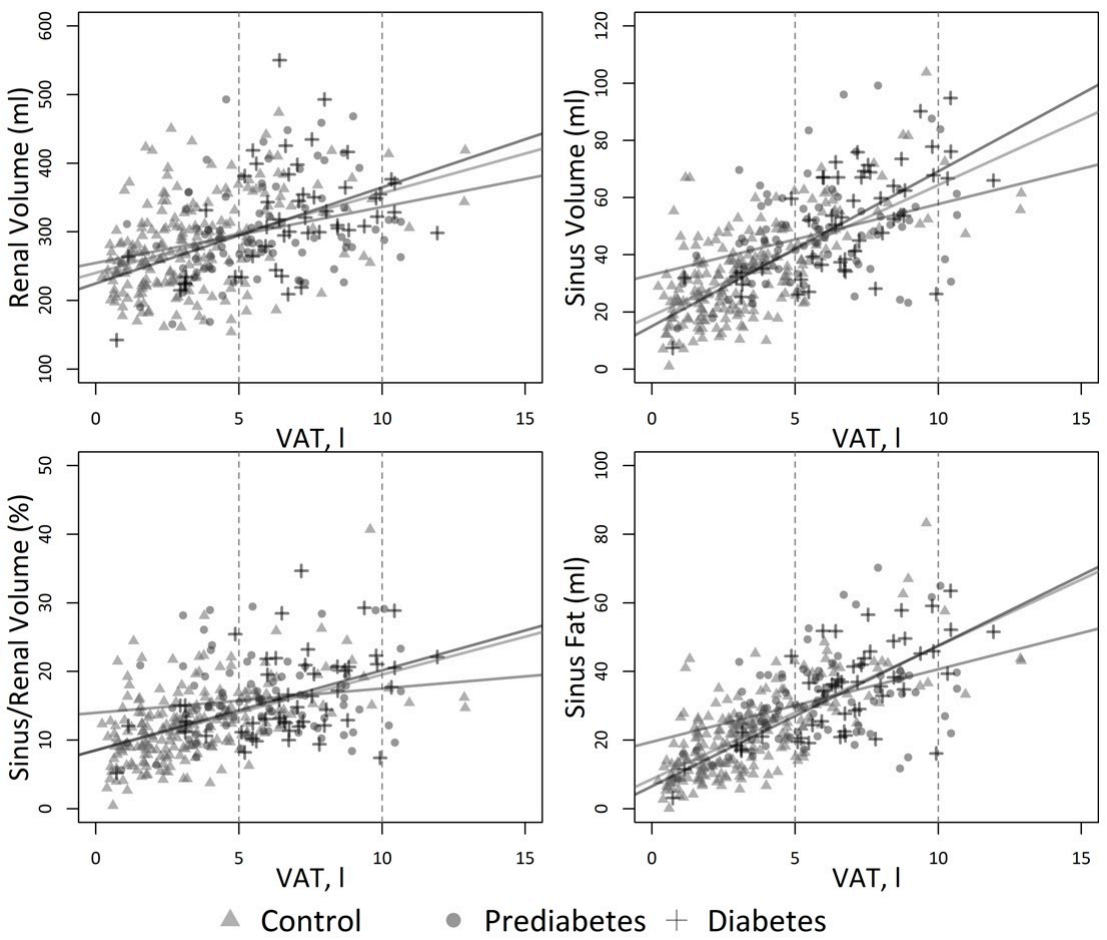
Scatter diagrams showing the correlation of the VAT with the glycemic groups. There was a significant correlation between VAT and renal sinus fat particularly for healthy controls and individuals with diabetes.

When stratifying according to glycemic status, there was also a significant correlation between VAT and sinus fat in normoglycemic individuals and individuals with diabetes (between r = 0.66 and 0.73) and a lower but still significant correlation in individuals with prediabetes (Table 5 and Fig 4) (between r = 0.35 and 0.40).

**Table 5:** Pearson’s correlation coefficients of VAT and renal volumes with corresponding 95% CI stratified by glycemic status.

	Renal Volume, ml	Sinus Volume, ml	Sinus Fat Component, ml
Control	0.42 [0.30, 0.52]	0.67 [0.59, 0.73]	0.73 [0.66, 0.78]
Prediabetes	0.28 [0.07, 0.46]	0.35 [0.15, 0.52]	0.40 [0.21, 0.57]
Diabetes	0.43 [0.17, 0.63]	0.66 [0.47, 0.80]	0.69 [0.51, 0.82]

## Discussion

As hypothesized, total renal compartment volumes significantly increased with glucose intolerance. Particularly renal sinus fat shows a considerable and significant increase in subjects with prediabetes compared to healthy controls. However, renal sinus fat is not independently associated with glycemic status and shows a strong correlation with VAT.

Assessment of kidney size and volume in the context of chronic kidney disease associated with cardiovascular risk profiles has been of long-standing interest with contradictory results [27–31]. In diabetic nephropathy pre-clinical studies show an increase of kidney volume even preceding hyperfiltrative stages [32–34]. although this could not be verified in a small case study [35]. Similarly to our study, the Framingham Heart Study has shown an increasing total kidney volume in diabetic individuals with normal GFR, while decreased kidney volume was associated with hypertension and reduced GFR [7]. In renovascular disease, kidney and cortex volume had a predictive value on clinical outcome [29, 30]. A recent study has shown a negative correlation of the kidney volume to the extent of chronic disease [36]. However, in our study cohort GFR was only slightly reduced in our well-adjusted study subjects with prediabetes and diabetes, so that extent of chronic kidney disease was only low and that a direct link of renal volume alteration to renal parenchymal disease cannot be deduced from this study.

Renal sinus fat has become of increasing interest when studying cardiovascular risk factors in metabolic syndrome, as perivascular adipose tissue forms an important multiorgan link between obesity, hepatic function, insulin resistance and macro- as well as microangiopathy [9, 23]. It is thought to obstruct lymph and blood outflow of the kidney, leading to increasing organ size. A recent study has shown that in a metabolically benign condition, renal sinus fat reduces the release of (pro)-inflammatory factors. In a metabolically malignant condition fatty liver-derived hepatokines, such as Fetuin-A act on human renal sinus fat. Thus, the beneficial influence on glomerular cells is abolished, possibly leading to renal dysfunction and damage [12]. Previous studies have demonstrated the presence of interindividually varying amounts of fat around the vessels of the renal hilum in humans potentially influencing renal/glomerular function via organ crosstalk [8, 13].

Renal sinus fat volume is associated with the number of prescribed antihypertensive medications and stage II hypertension [11] and is also thought to be an independent risk indicator of coronary artery calcification in middle-aged patients [37]. Our results corroborate with these findings, showing that individuals with prediabetes already show a considerable increase in renal sinus fat, whereas GFR remained almost constant. Renal sinus fat may also play an early role in the pathogenesis of exercise-induced albuminuria independently of sex, age, VAT and mean arterial peak pressure [13], so that it may serve as an early imaging biomarker for potential renal disease. Accordingly, in a large subcohort of the Framingham study, quantification of renal sinus fat accumulation was independently associated with both hypertension and chronic kidney disease [8]. In our study, there was a significant association between increasing urine albumin levels and renal sinus volume, but not renal sinus fat volume, so that we cannot provide a final conclusion to this point.

A cross-sectional study [38] found deposition of adipose tissue particularly into the left renal sinus, which was related with the VAT amount. However, reductions in VAT volume were not accompanied by reductions in renal sinus fat accumulation. An increasing number of studies suggest, that renal sinus fat plays an important role in obesity-induced renal injury [39] which could be diagnosed and linked with early biomarkers of kidney injury [38].

In our study, the volume of renal sinus fat was not independently associated with glycemic status and this association was not significant when corrected for cardiovascular risk factors. Interestingly, there was a strong correlation with VAT, explaining the major variability of renal sinus fat. These findings show, that both VAT and renal sinus fat may show interactions as perivascular adipose tissue, similarly to pericardial and hepatic fat. Our findings are similar to another study investigating the same study cohort, showing that pancreatic fat content differs significantly between subjects with prediabetes, diabetes and controls, but that association is confounded by age, gender, and the amount of VAT [18].

Additionally, it has been shown that not only renal sinus fat, but also an increase in intrarenal lipids could be detected in diabetes [40] and obesity associated nephropathy [41]. There is emerging evidence that these ectopic lipid-accumulation causes structural and functional changes of mesangial cells, podocytes, and proximal tubular cells and is associated with renal hypoxia [42]. The exploited two-point-Dixon-VIBE-sequence would principally allow for such intrarenal lipid quantification, however these results are only consistent, if fat content is above ten percent due to reduced noise effects, so that variation bias was too high (data not shown). More precise multi-echo-Dixon-VIBE-sequences such as used in other studies studying intrarenal lipids [40] were acquired in this study cohort, but were centered on the liver, so that the kidneys were only partially covered. Alternatively, magnetic resonance spectroscopy may be a reliable tool to study intrarenal lipids [43].

Our data was based on semi-automated segmentation and voxel-based volumetry of T1w-Dixon images. We chose a semi-automated approach as manual segmentation of abdominal organs is complex and tedious and is also prone to inter- and intraindividual bias [44]. Semi-automated segmentation and volumetry of the entire kidneys based on voxel summation is a robust method to assess discrete changes of organ volume [7], which may be overlooked by a manual approach as performed in previous studies [24, 45]. Our exploited algorithm was based on thresholding and geometrical approaches and did not comprise neural networks and deep learning approaches, so that manual correction was still required. However, total renal volume did only show a small inter-reader variability, whereas there was a larger relative variability for renal sinus fat, but still considerably smaller than the difference between healthy and prediabetic subjects.

There are several limitations to our study. First, our semi-automated algorithm did not satisfactory separate renal cortex and medulla (data not shown). As our focus lay on the assessment total renal volume and renal sinus fat, we did not further pursue corticomedullary discrimination and segmentation. Although we adjusted our analysis for variables that could serve as potential confounders of the relation between renal sinus fat and glycemic status, we are aware that this adjustment is always incomplete and confounding bias can never be fully excluded. Given that all participants in our study were Caucasian, the influence of ethnicity cannot be assessed. Data on the participants’ hydration status was not available in our study.

Our algorithm required manual correction, so that further refinement would be necessary to assess large volume cohort studies such as the German National Cohort [46] or UK Biobank [20] with up to 100,000 study subjects.

Lastly, in multiple regression models, beta coefficients from the same model are usually not corrected for multiple testing, as they do not denote independent tests [47]. Hence, we did not use the derived p-values for variable selection or make claims about the predictive ability of specific variables. Therefore, we have used the nominal alpha level of 0.05 and present all results from these three models without adjusting for the three independent tests.

In conclusion, renal volume and particularly renal sinus fat volume already increases significantly in prediabetic subjects and is significantly associated with VAT. This shows, that renal sinus fat is a perivascular adipose tissue, which early undergoes changes in the development of metabolic disease. Our findings underpin that that renal sinus fat is a link between metabolic disease and associated chronic kidney disease, making it a potential imaging biomarker when assessing perivascular adipose tissue.

## Supporting information

S1 File (DOCX)
