# Supplementary material for: Renal and renal sinus fat volumes as quantified by magnetic resonance imaging in subjects with prediabetes, diabetes, and normal glucose tolerance
Source: PLoS One. 2020 Feb 19;15(2):e0216635. doi: 10.1371/journal.pone.0216635 (PMC7029849; doi:10.1371/journal.pone.0216635)
Supplement: S1 File — (DOCX) [file pone.0216635.s001.docx]

|  | Renal Volume (ml) | | | | |  | Sinus Volume (ml) | | | | |  | Sinus Fat Component (ml) | | | | |  |
| --- | --- | --- | --- | --- | --- | --- | --- | --- | --- | --- | --- | --- | --- | --- | --- | --- | --- | --- |
|  | β | β | 95%-CI | 95%-CI | P-Value | P-Value | β | β | 95%-CI | 95%-CI | P-Value | P-Value | β | β | 95%-CI | 95%-CI | P-Value | P-Value |
| Age, Years | 0.7 | 6.8 | [-6.3, 7.7] | [-0.3, 13.8] | 0.840 | 0.059 | 2.6 | 2.8 | [0.7, 4.4] | [0.9, 4.7] | 0.006 | 0.004 | 2.3 | 2.5 | [1.1, 3.6] | [1.2, 3.8] | <0.001 | 0.000 |
| VAT | 10.1 | 9.7 | [1.6, 18.7] | [1.5, 17.9] | 0.021 | 0.020 | 7.5 | 7.5 | [5.3, 9.7] | [5.3, 9.7] | <0.001 | 0.000 | 6.0 | 6 | [4.5, 7.5] | [4.4, 7.5] | <0.001 | 0.000 |
| Height |  | 22.6 |  | [14.8, 30.4] |  | 0.000 |  | 1 |  | [-1.1, 3.1] |  | 0.343 |  | 0.7 |  | [-0.8, 2.2] |  | 0.358 |
| HDL | -10.5 | -10.5 | [-16.8, -4.2] | [-16.5, -4.4] | 0.001 | 0.001 | 0.2 | 0.2 | [-1.4, 1.8] | [-1.4, 1.8] | 0.799 | 0.796 | 0 | -0 | [-1.2, 1.1] | [-1.2, 1.1] | 0.948 | 0.951 |
| LDL | -7.6 | -6.2 | [-13.0, -2.2] | [-11.4, -1.0] | 0.006 | 0.020 | -1.3 | -1.3 | [-2.7, 0.1] | [-2.7, 0.1] | 0.061 | 0.076 | -0.5 | -0.4 | [-1.5, 0.5] | [-1.4, 0.5] | 0.329 | 0.376 |
| Albumin (Urine) | 2.2 | 2.2 | [-3.1, 7.6] | [-3.0, 7.3] | 0.414 | 0.411 | 1.6 | 1.6 | [0.2, 3.0] | [0.2, 3.0] | 0.025 | 0.026 | 0.5 | 0.5 | [-0.5, 1.4] | [-0.5, 1.4] | 0.342 | 0.345 |
| Liver Fat | 0.2 | 1.6 | [-7.2, 7.5] | [-5.5, 8.7] | 0.967 | 0.653 | 1.6 | 1.6 | [-0.3, 3.5] | [-0.3, 3.5] | 0.109 | 0.096 | 1.6 | 1.6 | [0.3, 2.9] | [0.3, 3.0] | 0.020 | 0.017 |
| GFR | 23.1 | 25 | [16.4, 29.7] | [18.6, 31.4] | <0.001 | 0.000 | 3.0 | 3 | [1.3, 4.7] | [1.3, 4.8] | <0.001 | 0.001 | 2.1 | 2.1 | [0.9, 3.3] | [0.9, 3.3] | <0.001 | 0.001 |
| Sex, female | -57.6 | -23.2 | [-70.6, -44.7] | [-40.4, -6.0] | <0.001 | 0.008 | -8.8 | -7.2 | [-12.1, -5.5] | [-11.9, -2.6] | <0.001 | 0.002 | -7.3 | -6.3 | [-9.7, -5.0] | [-9.5, -3.0] | <0.001 | 0.000 |
| Hypertonia, yes | 10.1 | 11.2 | [-2.6, 22.8] | [-0.9, 23.4] | 0.118 | 0.071 | 3.7 | 3.7 | [0.4, 6.9] | [0.5, 7.0] | 0.027 | 0.026 | 3.0 | 3 | [0.7, 5.2] | [0.7, 5.3] | 0.012 | 0.011 |
| Prediabetes | 11.3 | 10 | [-2.6, 25.2] | [-3.3, 23.3] | 0.110 | 0.140 | 4.0 | 4 | [0.4, 7.6] | [0.4, 7.6] | 0.028 | 0.030 | 1.7 | 1.7 | [-0.8, 4.3] | [-0.8, 4.2] | 0.172 | 0.182 |
| Diabetes | 7.7 | 10.2 | [-11.1, 26.5] | [-7.8, 28.2] | 0.421 | 0.268 | 0.0 | 0.1 | [-4.8, 4.8] | [-4.7, 5.0] | 1.000 | 0.964 | -1.7 | -1.6 | [-5.1, 1.7] | [-5.0, 1.8] | 0.324 | 0.347 |
